# Chronic nicotinamide riboside supplementation is well-tolerated and elevates NAD^+^ in healthy middle-aged and older adults

**DOI:** 10.1038/s41467-018-03421-7

**Published:** 2018-03-29

**Authors:** Christopher R. Martens, Blair A. Denman, Melissa R. Mazzo, Michael L. Armstrong, Nichole Reisdorph, Matthew B. McQueen, Michel Chonchol, Douglas R. Seals

**Affiliations:** 1 Department of Integrative Physiology, University of Colorado Boulder, Boulder, CO USA; 2 Department of Pharmaceutical Sciences, Skaggs School of Pharmacy and Pharmaceutical Sciences, University of Colorado Anschutz Medical Campus, Denver, CO USA; 3 Division of Renal Diseases and Hypertension, University of Colorado Anschutz Medical Campus, Denver, CO USA

## Abstract

Nicotinamide adenine dinucleotide (NAD^+^) has emerged as a critical co-substrate for enzymes involved in the beneficial effects of regular calorie restriction on healthspan. As such, the use of NAD^+^ precursors to augment NAD^+^ bioavailability has been proposed as a strategy for improving cardiovascular and other physiological functions with aging in humans. Here we provide the evidence in a 2 × 6-week randomized, double-blind, placebo-controlled, crossover clinical trial that chronic supplementation with the NAD^+^ precursor vitamin, nicotinamide riboside (NR), is well tolerated and effectively stimulates NAD^+^ metabolism in healthy middle-aged and older adults. Our results also provide initial insight into the effects of chronic NR supplementation on physiological function in humans, and suggest that, in particular, future clinical trials should further assess the potential benefits of NR for reducing blood pressure and arterial stiffness in this group.

## Abstract (web summary)

Declining NAD^+^ levels have been linked to aging-associated pathologies. Here the authors present results of a double-blind, randomized crossover trial on 30 healthy middle-aged individuals to show that nicotinamide riboside effectively elevates NAD^+^ levels in humans, appears to be well tolerated, and may have potential to improve cardiovascular parameters.

## Introduction

Advancing age is the primary risk factor for the development of cardiovascular disease (CVD), which remains the leading cause of morbidity and mortality in industrial and post-industrial societies^1^. The increase in CVD risk with aging is driven largely by adverse changes to arteries, including stiffening of the aorta, and by increases in systolic blood pressure^2^. As such, interventions designed to lower blood pressure and/or improve arterial function hold promise for preventing age-related CVD.

Chronic calorie restriction (CR) prevents the development of arterial dysfunction and increases in blood pressure with aging in rodents^3,4^, and lowers arterial stiffness and blood pressure in overweight-obese middle-aged and older adults^5,6^. Despite numerous health benefits, adherence to chronic CR remains poor and possibly even unsafe in normal weight older adults^7–9^. As such, there is a critical need to establish safe, practical alternatives to regular CR for enhancing cardiovascular function and health with aging in humans^10^.

The recent identification of several key molecular mechanisms responsible for CR-mediated longevity in model organisms has led to an exciting search for “CR-mimetic” interventions to improve cardiovascular and other physiological functions with aging^11,12^. In this regard, nicotinamide adenine dinucleotide (NAD^+^) has emerged as a critical signaling molecule and essential substrate for sirtuins, a class of enzymes that mediate several of the beneficial effects of CR in model organisms^13,14^, including the maintenance of cardiovascular function^15^. Moreover, CR has been shown to increase NAD^+^ levels in pre-clinical models^16,17^. The cellular bioavailability of NAD^+^ and related metabolites declines in animals and in humans during normal aging^13,18–21^ and may contribute to physiological aging by reducing sirtuin activity. Although NAD^+^ can be synthesized de novo from the amino acid tryptophan, this process does not occur in all tissues, requiring most cells to rely on a salvage pathway for regenerating NAD^+^ from other intracellular intermediates, which are primarily made available through dietary sources^22^. Vitamin B_3_ (niacin: i.e., nicotinic acid and nicotinamide) enters this salvage pathway and acts as a NAD^+^ precursor; however, nicotinic acid is associated with undesirable flushing at therapeutic doses^23^ and nicotinamide does not reliably activate (and may even inhibit) sirtuins despite raising concentrations of NAD^+^^24–26^. Therefore, administration of nicotinic acid or nicotinamide is unlikely to be widely adopted for maintaining health and function with aging.

In contrast to these compounds, oral supplementation with either of the NAD^+^ metabolites, nicotinamide mononucleotide (NMN) or nicotinamide riboside (NR), increases levels of NAD^+^ and improves multiple physiological functions in animal models^18,27,28^. Indeed, we recently demonstrated that supplementation of NMN in the drinking water improved cardiovascular function in old mice^29^. Moreover, CR increases concentrations of NR and NAD^+^ and restores normal circadian gene transcription in the liver, further suggesting that NR may act as a CR mimetic^30^. Thus, NMN and NR are NAD^+^ boosting compounds that hold promise for enhancing cardiovascular health and physiological function with aging^31,32^.

Despite these encouraging results from preclinical studies, the tolerability and effectiveness of chronic supplementation with NMN or NR have not been established in humans. Because NR is readily taken up by cells and acts as a direct vitamin precursor for NAD^+^ synthesis^33^, its recent development as a dietary ingredient (NIAGEN^®^, ChromaDex Inc., Irvine, CA) has provided the first opportunity to translate the potential benefits of NAD^+^ boosting molecules to people. In this regard, a recent study showed that single doses of NR stimulated blood cellular NAD^+^ metabolism in healthy humans in a dose-dependent manner^26^. However, the tolerability of chronic NR supplementation and its efficacy for increasing NAD^+^ bioavailability have not been established in humans, and we lack even initial insight into the potential of NR for improving cardiovascular and other physiological functions with human aging.

To address these important research gaps, we conducted a small randomized, placebo-controlled, crossover clinical trial of NR supplementation (500 mg, 2×/day) to assess its overall tolerability and efficacy vs. placebo for raising levels of NAD^+^-related metabolites in healthy middle-aged and older men and women. We also took the opportunity to gain preliminary insight into the effects of chronic NR supplementation for improving cardiovascular and other physiological functions associated with risk of clinical diseases and/or disability with aging. Our results demonstrate that 6 weeks of NR supplementation at this dose is well-tolerated in humans and effectively increases blood cellular NAD^+^ concentrations. Exploratory analyses of the effects of chronic NR supplementation on physiological function in this cohort of healthy middle-aged and older adults suggest that the potential for reducing systolic blood pressure and arterial stiffness may be the most promising hypotheses to investigate in future larger-scale clinical trials, particularly in individuals with elevated baseline blood pressure.

## Results

### Subject enrollment and baseline characteristics

**Fig. 1:**
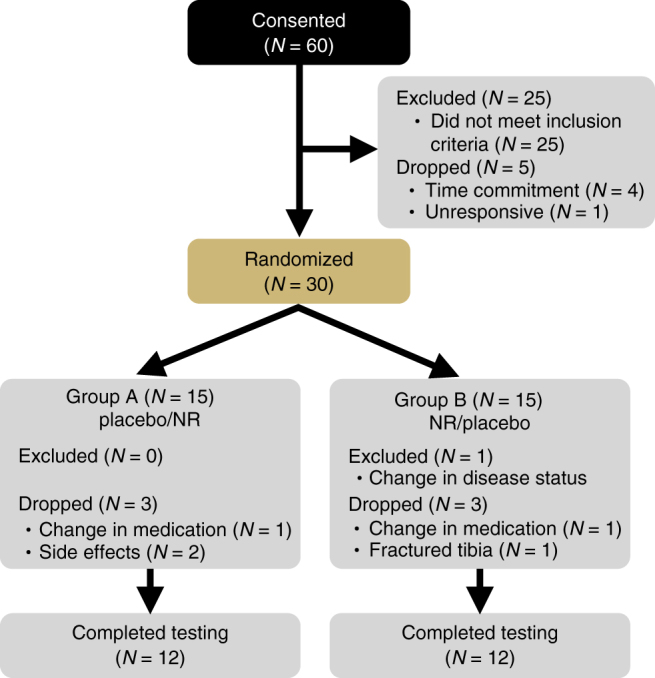
Study flow diagram

Information on subject consent, randomization, testing and completion is presented in Fig. 1. Sixty healthy middle-aged and older men and women between the ages of 55 and 79 years were consented for this study, which was registered on clinicaltrials.gov under the identifier NCT02921659 and conducted between March 2015 and September 2016. The individuals recruited for this study were lean (average BMI = 24 ± 4 kg m^−2^) and healthy, and were representative of the late middle-aged/older adult population within the greater Boulder County Colorado community. Twenty-five participants did not meet inclusion criteria and were excluded without being randomized. Four participants dropped out of the study prior to randomization due to a conflict with time commitment, and one individual was unresponsive to scheduling requests, resulting in a total of 30 subjects remaining for randomization. Of these, 15 subjects were randomized to Group A, which received placebo capsules during the first 6 weeks of the study before crossing over to receive NR capsules for the remaining 6 weeks. The other 15 subjects were randomized to Group B, which received NR capsules first followed by placebo. One subject was withdrawn from Group A due to a change in medication status that no longer met inclusion criteria, and two subjects in Group A elected to drop out of the study due to a complaint of side effects (see below). Two subjects were withdrawn from Group B due to a change in health or medication status that no longer met inclusion criteria, and one subject elected to drop out of Group B due to a non-study-related injury, resulting in a total of 24 subjects who completed the trial. Removal of these six subjects did not influence the overall makeup of the group because the characteristics for the 24 subjects who completed the trial were similar to those for all 30 subjects who were initially randomized (Supplementary Table 8). The subjects that completed the study were well matched between groups for age, sex and clinical characteristics, and all baseline values were within normal clinical ranges (Table 1).

**Table 1:** Baseline subject characteristics

Subject characteristic	Group A	Group B	All subjects combined
Sex (M/F)	5/7	6/6	11/13
Age (years)	64 ± 6	66 ± 9	65 ± 7
Mass (kg)	67 ± 16	69 ± 14	68 ± 15
BMI (kg m^−2^)	23 ± 4	24 ± 3	24 ± 4
Total body fat (%)	27 ± 10	29 ± 11	28 ± 10
Systolic blood pressure (mmHg)	122 ± 18	120 ± 17	121 ± 17
Diastolic blood pressure (mmHg)	77 ± 11	72 ± 9	74 ± 10
Fasting glucose (mg dl^−1^)	89 ± 8	87 ± 8	88 ± 8
Total cholesterol (mg dl^−1^)	192 ± 38	183 ± 36	187 ± 36
HDL cholesterol (mg dl^−1^)	69 ± 12	69 ± 25	69 ± 19
LDL cholesterol (mg dl^−1^)	107 ± 36	94 ± 23	101 ± 30

Includes all subjects who completed the study (*N* = 24) by randomization group (A = placebo, NR; B = NR, placebo) and in all subjects combined. Data are mean ± SD.

*BMI* body mass index, *HDL* high-density lipoprotein, *LDL* low-density lipoprotein

### Treatment-emergent adverse events

Adherence to the study treatments was excellent, with all subjects consuming greater than 95% of all NR and placebo capsules administered. NR was well tolerated at the dose tested, and no serious adverse events occurred. A total of 14 treatment-emergent adverse events (AEs) were reported by 7 of the 30 participants enrolled in the study, with the other 23 subjects reporting no AEs. All self-reported AEs were mild in severity. The reported symptoms included nausea, flushing, leg cramps and increased bruising during the NR condition, and headache, skin rash, flushing, fainting and drowsiness during the placebo condition (Table 2*)*. Only 2 out of the 30 enrolled subjects (<10%) dropped out of the study due to a complaint of side effects, both occurring while subjects were in the placebo phase (headache and skin rash); no subject dropped out during the NR treatment condition.

**Table 2:** Treatment-emergent adverse events (AEs)

Adverse event (AE)	Placebo No. of events (no. of events/patient)	NR No. of events (no. of events/patient)
Headache	4 (1)	0 (0)
Nausea	0 (0)	1 (1)
Skin rash	1 (1)	1 (1)
Flushing/Hot flashes	2 (1)	1 (1)
Fainting	1 (1)	0 (0)
Drowsiness	1 (1)	0 (0)
Leg cramps	0 (0)	1 (1)
Increased bruising	0 (0)	1 (1)

Data represent number (*n*) of times AE was reported. Number of subjects reporting AEs (*n* = 7); Number of subjects reporting ≥2 AE (*n *= 5)

Obtained from self-report during bi-weekly check-in visits over each phase. Based on *N* = 30 randomized subjects

Clinical laboratory values were obtained from blood samples collected at the end of each treatment phase in 21 of the 24 subjects who completed the study. Complete blood work could not be obtained from the remaining three subjects due to failed catheterization (*n* = 1), administrative error (*n* = 1) or study nurse error (*n *= 1). No meaningful differences were observed between treatment conditions for hematology (Supplementary Table 1), blood chemistry, including markers of renal function and liver enzymes (Supplementary Table 2), or blood lipid profiles (Supplementary Table 3). Importantly, all clinical laboratory values remained within the normal reference range during both the placebo and NR conditions. Collectively, these results indicate that oral supplementation with NR for 6 weeks at this dose is well-tolerated in healthy middle-aged and older adults.

### Efficacy of NR for increasing NAD^+^ and related metabolites

After demonstrating the tolerability of chronic NR supplementation, our primary objective was to determine if NR raises blood cellular NAD^+^ metabolism in humans. Because blood NAD^+^ and several related metabolites of interest have recently been shown to be measurable in circulating peripheral blood mononuclear cells (PBMCs), but undetectable in plasma and urine^26^, we assessed the NAD^+^ metabolome in circulating PBMCs, as previously established^34^.

**Fig. 2:**
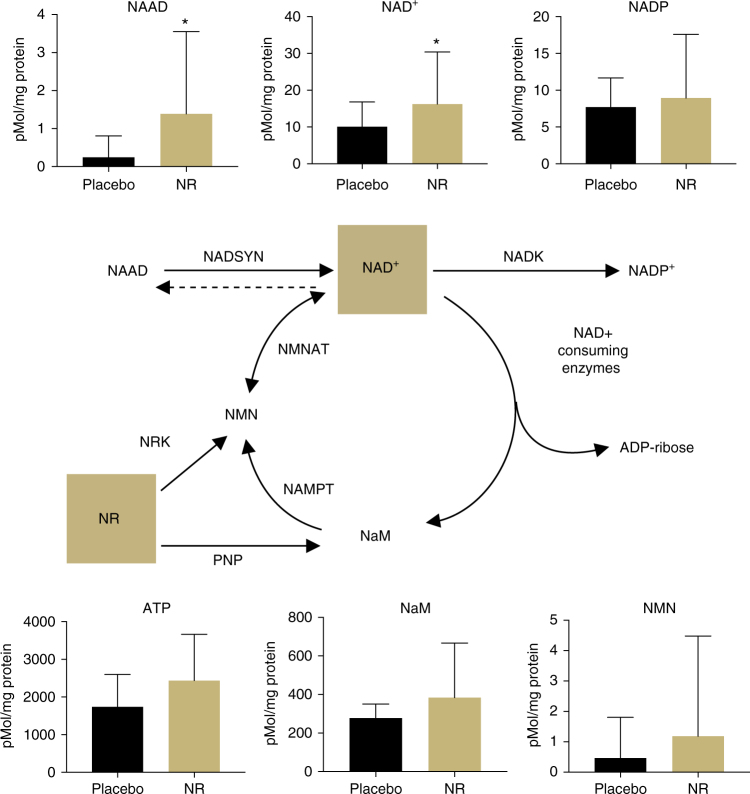
NAD^+^ metabolome. NAD^+^and related metabolite concentrations in peripheral blood mononuclear cells increased after oral placebo vs. NR supplementation normalized to total protein content. Data are mean ± SD. * indicates unadjusted *P* < 0.05 by one-tailed paired *t-*test. *N* = 21 (Group A = 11; Group B = 10)

Oral NR supplementation effectively elevated levels of NAD^+^ in PBMCs by ~60% compared with placebo (mean change = 6.2 pmol per mg protein; one-sided 95% CI (0.074, ∞)). The mean level of NADP^+^ also increased, but did not reach statistical significance (mean change = 1.2 pmol per mg protein; one-sided 95% CI (−2.15, ∞)) (Fig. 2 and Table 3). Of note, NR also elevated levels of nicotinic acid adenine dinucleotide (NAAD) nearly fivefold above the placebo condition (mean change = 1.1 pmol per mg protein; 95% CI (0.26, ∞)), confirming a previous report that NAAD is a highly sensitive and reliable biomarker of increased NAD^+^ metabolism and a product of NR utilization in humans^26^. NR also elevated the mean concentration of nicotinamide (NaM), but this was not statistically significant (mean change = 106.5 pmol per mg protein; one-sided 95% CI (−10.03, ∞)). An increase in NaM would suggest an increase in the activity of NAD^+^-consuming enzymes, which catalyze the breakdown of NAD^+^ into NaM and ADP-Ribose^35^. Though not significant, we also observed an ~1.5-fold increase in NMN (mean change = 0.72 pmol per mg protein; one-sided 95% CI (−0.60, ∞)), which may indicate the possible conversion of NR to NMN by nicotinamide riboside kinase (NRK) enzymes or further metabolism of NaM into NMN by nicotinamide phosphoribosyltransferase (NAMPT)^35^. Consistent with the only other report of NR ingestion in humans^26^, we were unable to detect NR concentrations in PBMCs during either treatment condition, despite using optimized recovery methods. The magnitude by which NAD^+^ increased in response to NR supplementation was negatively associated with blood cellular NAD^+^ concentration during the placebo condition (*R* = −0.49, *R*^2^ = 0.25), suggesting a greater response in individuals with naturally low blood cellular NAD^+^ levels.

**Table 3:** NAD^+^metabolites

Metabolite	Median		Range		*P*-value
	Placebo	NR	Placebo	NR	
NAAD	0.0	0.0	0.0−2.3	0.0−8.7	0.018*
NAD^+^	7.7	12.2	0.0−27.4	4.7−67.8	0.048*
NADP	6.1	6.3	3.3−17.9	2.7−42.7	0.267
NaM	257.6	278.6	109−411	171−1357	0.065
NMN	0.0	0.0	0.0−5.5	0.0−11.9	0.179
ATP	1592	2205	363−3446	763−5459	0.032

All values expressed as pmol per mg protein. * represents unadjusted *P* < 0.05; ATP represents secondary outcome assessed at Bonferroni-adjusted *P* < 0.006

In addition to boosting NAD^+^-specific metabolites in PBMCs, we also observed increases in the mean concentration of other metabolites involved in the regulation of energy production and metabolism, including adenosine and adenosine triphosphate (ATP; mean change = 699 pmol per mg protein; one-sided 95% CI (84, ∞); Fig. 2 and Table 3); however, analysis of this metabolite was considered a secondary outcome and the increase did not attain statistical significance after correction for multiple comparisons. NR supplementation also tended to raise levels of adenosine diphosphate (ADP) and adenosine monophosphate (AMP), though increases in these metabolites did not reach statistical significance (Supplementary Table 4). Collectively, these findings indicate that chronic NR supplementation effectively stimulates NAD^+^ metabolism in healthy middle-aged and older men and women.

### Effect of NR on indicators of cardiovascular health

**Fig. 3:**
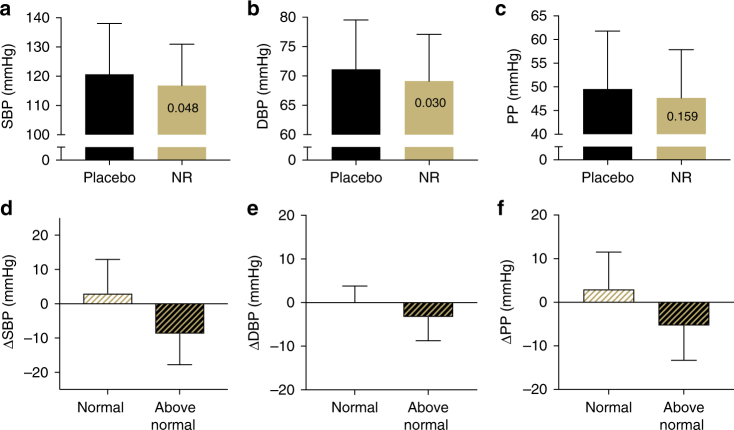
Blood pressure. Effect of 6 weeks of oral placebo vs. NR supplementation on **a** systolic (SBP) and **b** diastolic (DBP) blood pressure, and **c** pulse pressure (PP) in healthy middle-aged and older adults as a whole *N* = 24 (Group A = 12; Group B = 12), and overall change from placebo in blood pressure parameters (**d−f**) in subjects with normal (*N* = 11) vs. above normal (*N* = 13) baseline BP. Data are mean ± SD. *P-*values reported in individual bars based on a one-tailed paired *t*-test (panels **a**−**c** only) and an adjusted alpha level set at 0.006

Supplementation with NR tended to lower mean systolic (SBP; mean change = −3.9 mmHg; one-sided 95% CI (−∞, −0.058)) and diastolic (DBP; mean change = −2.0 mmHg; one-sided 95% CI (−∞, −0.26)) blood pressure (BP) in all subjects as a group (Fig. 3a–c); however, these comparisons were not statistically significant after correction for multiple comparisons. Because the risk of cardiovascular events is greatly increased in individuals with above-normal baseline BP^36^, we performed a follow-up analysis to compare the effect of NR on BP in the participants with BP in the normal range (SBP/DBP < 120/80 mmHg; *N* = 11) vs. those with BP in the elevated/stage I hypertension range (SBP, 120–139 mmHg; DBP, 80–89 mmHg; *N* = 13) based on recently updated guidelines^37^. Of particular note, mean SBP was 9 mmHg lower after NR vs. placebo in individuals with elevated/stage I hypertension, whereas no change was observed in subjects with initial SBP in the normal range (Fig. 3d). Because this post-hoc subgroup analysis was exploratory, no statistical inferences can be made. The median values and ranges for all blood pressure variables are provided in Supplementary Table 6.

**Fig. 4:**
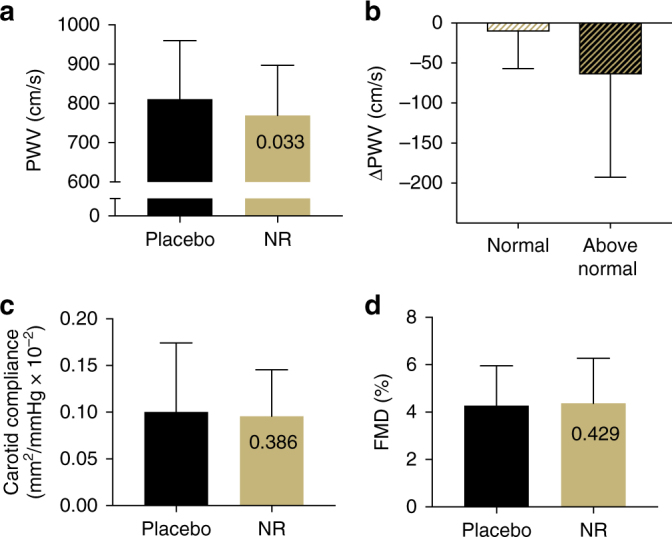
Arterial function. Effect of 6 weeks of oral placebo vs. NR supplementation on **a** aortic pulse wave velocity (PWV) as a whole (*N* = 24; 12 per group), **b** subgroups of individuals with normal (*N* = 11) vs. above-normal (*N* = 13) baseline BP); **c** carotid artery compliance (CC) and **d** brachial artery flow-mediated dilation (FMD) in the overall groups (*N* = 24; 12 per group). Data are mean ± SD. *P*-values reported in individual bars based on one-tailed paired *t*-test (panels **a**, **c**, and **d** only) and an adjusted alpha level set at 0.006

We also observed a trend towards a reduction in the mean carotid-femoral pulse wave velocity (PWV) with NR supplementation, the clinical “gold standard” measure of the stiffness of the aorta^38^, and a strong independent risk factor for incident cardiovascular events with aging and age-related diseases (Fig. 4a*;* mean change = −41.5 m s^−1^; one-sided 95% CI (−∞, −4.8)). However, this reduction was not statistically significant after correction for multiple comparisons. Similar to our exploratory analysis of BP, NR supplementation tended to lower aortic stiffness (carotid-femoral PWV) more in individuals with higher baseline BP (Fig. 4b), although no statistical inferences were made for this post-hoc comparison. No effect of NR was observed on ultrasound-determined carotid artery compliance (Fig. 4c) or brachial artery flow-mediated dilation, a measure of vascular endothelial function (Fig. 4d).

### Effect of NR on other domains of physiological function

To gain exploratory insight into potential benefits of NR supplementation on other domains of physiological function in healthy middle-aged and older adults, we assessed a wide variety of outcomes indicative of metabolic function, motor function, and exercise capacity/performance. Total energy intake and expenditure, oxidative fuel source (carbohydrate vs. fat), and physical activity patterns were not affected by NR (Supplementary Table 7). Likewise, we observed no difference in body mass, body mass index (BMI) or percent body fat compared with the placebo arm (Supplementary Table 7) and no differences were observed in measures of glucose or insulin regulation (Supplementary Table 7). Finally, there was no effect of the intervention on overall motor function (Supplementary Figure 2), maximal exercise capacity, as assessed by VO_2_ max and treadmill time to exhaustion (Supplementary Figure 1A, B), or on markers of submaximal exercise performance (Supplementary Figure 1C−F).

## Discussion

The primary finding of this study is that chronic oral supplementation with 1000 mg per day of NR is a well-tolerated and effective strategy for stimulating NAD^+^ metabolism in healthy middle-aged and older humans. Additionally, our exploratory analyses of the effects of NR supplementation on physiological function suggest that the ability of NR to reduce SBP and aortic stiffness, two clinically important risk indicators of cardiovascular function and health, are among the most promising hypotheses to test in a future larger-scale clinical trial, particularly in individuals with above-normal baseline SBP.

In this small initial intervention trial, NR was well tolerated and elicited no serious adverse effects. Additionally, we found that NR stimulated NAD^+^ metabolism without any difference in treatment-emergent AEs compared with placebo, supporting previous suggestions that NR may be a more suitable NAD^+^ precursor than niacin (i.e., nicotinic acid and nicotinamide), which` also is capable of entering the NAD^+^ salvage pathway, but is associated with a painful flushing sensation at therapeutic doses^22,23^. The flushing response induced by niacin is specifically caused by the binding of nicotinic acid to the Gpr109A receptor on epithelial cells^39,40^, an action that is not expected to occur with NR, thereby minimizing the risk of this side effect at equivalent doses^22^. Although three of the subjects in our study did report flushing, two of these subjects were taking the placebo capsules, suggesting that NR itself did not appear to be associated with flushing. Despite these promising findings, we wish to emphasize that the size of the cohort in the present study is insufficient to establish the broader safety profile of NR at this dose. Larger, more definitive clinical trials, similar to those conducted with niacin, will be necessary to confirm this preliminary evaluation of tolerability and to more definitively assess its overall safety. However, indirect evidence for the potential safety of NR supplementation is suggested by the widespread use of niacin over past 60 years for the treatment of high cholesterol, with limited side effects other than flushing^41^.

Niacin serves as important dietary precursor to NAD^+^ and helps sustain cellular function and protect against pellagra, a condition characterized by dark pigmented skin, dermatitis, diarrhea, and dementia. Like niacin, NR has been detected in cow’s milk^42,43^ and may theoretically act as another vitamin precursor form of NAD^+^. However, the amount of NR naturally consumed through the diet is likely much smaller than the dose tested in the present study. The primary natural sources of vitamin B_3_ come from NAD^+^, NADPH and NADH, which are more abundant in food sources and are broken down into salvageable precursors, including niacin and NR. To protect against pellagra, the recommended daily allowances of niacin for adult men and women have been set at 16 and 14 mg per day, respectively^22^, whereas higher doses of niacin have beneficial effects on lipid profiles of individuals at risk for cardiovascular events^23^. It is plausible that the daily requirement for NAD^+^ precursors may increase with advancing age due to decreasing NAD^+^ bioavailability, although no such recommendation presently exits.

An important goal of the present study was to identify clinically relevant physiological outcomes for future larger-scale (phase II) clinical trials of NR supplementation. The most promising result of these exploratory analyses was a trend towards an improvement in selective indicators of cardiovascular function. Compared with placebo, NR tended to lower SBP and aortic stiffness, two major independent risk factors for incident cardiovascular events and disease with advancing age^2,36^, in the overall group. A follow-up analysis suggested that this trend was most pronounced in individuals with baseline BP between 120 and 139 mmHg, a subgroup currently classified clinically as having either “elevated” SBP (120–129 mmHg) or stage 1 systolic hypertension (130–139 mmHg). The mean decrease in SBP after NR treatment in this subgroup approached 10 mmHg—a magnitude of change associated with a 25% decrease in incident CV events in a recent major anti-hypertensive drug trial in older adults^44^. If this magnitude of SBP reduction with NR supplementation is confirmed in a larger clinical trial, such an effect could have broad biomedical implications. SBP in this range (120−139 mmHg) is observed in ~50% of all middle-aged and older adults in the U.S.^45^ Moreover, SBP < 140 mmHg is responsible for at least one-third of all BP-attributable deaths^46^ and is associated with increased risk of heart disease, stroke, cognitive impairment/dementia and chronic kidney disease, among other disorders of aging^47^. Importantly, for individuals with SBP of 120–139 mmHg, lifestyle modifications, such as healthy diet and regular exercise are recommended before prescribing anti-hypertensive medications^37^. Given the low adherence to healthy lifestyle practices in middle-aged and older adults^7^, a natural, CR-mimicking, dietary supplement like NR with potential BP-lowering effects might represent a complementary approach for preserving cardiovascular health with aging.

We also observed a trend towards a reduction in carotid-femoral PWV after NR supplementation in the present study, suggesting that this measure of aortic stiffness may represent another promising cardiovascular outcome of interest for a larger future clinical trial. As with the effects on SBP, individuals with higher baseline BP appeared to demonstrate the largest mean changes in carotid-femoral PWV in response to NR treatment. Carotid-femoral PWV is not only independently predictive of incident CVD in older adults, but has recently been linked to numerous other age-associated diseases and disorders including mild cognitive impairment, chronic kidney disease, and frailty^48–51^. Thus, healthy lifestyle-mimicking dietary supplements with the potential to reduce age-related stiffening of the aorta would be of significant clinical interest. It should be noted that any NR-associated changes in BP may have contributed to corresponding changes in carotid-femoral PWV (and vice versa), so the changes in SBP and arterial stiffness are interrelated^52–54^.

Little is known about the underlying mechanisms by which NAD^+^ precursors may reduce BP and aortic stiffness in humans. NAD^+^ is an obligate substrate for the deacetylase sirtuin 1 (SIRT-1), which is implicated in the maintenance of healthy vascular function^3,15,55^. In this regard, treatment with the proposed pharmacological SIRT-1 activator SRT1720 protects against the development of aortic stiffness and hypertension in Klotho-deficient mice (a model of accelerated aging), by lowering vascular oxidative stress^56^. Likewise, we have demonstrated that supplementation with NMN reverses aortic stiffening in old mice to youthful levels by increasing aortic SIRT-1 activation and reversing age-related increases in aortic oxidative stress, collagen deposition and elastin fragmentation^29^. Based on these preclinical studies, it is possible that NR may similarly affect BP and aortic stiffness in humans through a mechanism involving SIRT-1 activation; however, future mechanistic studies are needed to test this and related hypotheses. Such studies will be technically challenging in humans, and it will be important to try to separate the effects of SIRT-1 activation from the likely pleotropic effects of boosting the NAD^+^ metabolome.

In addition to cardiovascular parameters, we also assessed the effect of NR on other domains of physiological function. Our findings suggest that a relatively short (6-week) intervention with NR did not change total energy expenditure or energy expenditure from fat oxidation (based on assessment of RER) at rest. We also did not observe any improvement in blood glucose control or insulin sensitivity. In both cases, it is important to note that this study was conducted in lean, healthy middle-aged and older adults without baseline metabolic dysfunction. These observations are in agreement with previous findings that NR modulates resting metabolic rate only in mice exposed to a high-fat diet^57^, and does not influence blood glucose regulation in animals fed normal chow diets^58^. Based on these preclinical observations, it is possible that chronic NR supplementation might improve metabolic function in groups who consume unhealthy diets and/or have metabolic disorders such as obesity, diabetes, and/or the metabolic syndrome.

Our results also suggest that NR does not obviously improve aerobic exercise capacity or motor function in healthy middle-aged and older men and women with good baseline physical status. Although Frederick et al.^28^ recently identified a role for NAD^+^ in rescuing neuromuscular function and exercise capacity in mice, these studies were conducted using a genetically induced model of impaired NAD^+^ bioavailability that may represent more severe NAD^+^ depletion than that which occurs with healthy aging. Future studies should explore the role of NR supplementation on aerobic exercise capacity and motor performance in groups with impaired mobility such as frail older adults and in individuals with chronic diseases associated with reduced cardiorespiratory fitness and exercise intolerance.

In summary, the results of the present study provide initial evidence that chronic NR supplementation is well-tolerated in healthy middle-aged and older adults, and extend recent findings that acute supplementation with NR is effective for stimulating NAD^+^ metabolism in humans. Furthermore, we provide the first insight into the effects of NR supplementation on physiological function in humans, and identify SBP and aortic stiffness as promising cardiovascular outcomes to be assessed in larger-scale clinical trials. Future work should also compare the relative increases in NAD^+^ and the physiological benefits of NR with conventional CR in order to further evaluate the use of this dietary supplement as a true “CR-mimetic” compound.

Finally, we wish to emphasize certain limitations of this initial trial on chronic NR supplementation in humans. Because the physiological outcomes in this study were designed to be exploratory in nature, the associated statistical inferences for those variables were based on one-sided hypothesis testing and the alpha level was set at a conservative *P* < 0.006 to account for multiple testing. More targeted studies (e.g., phase-II clinical trials) with fewer outcomes based on two-sided statistical inference are needed to confirm the effects of NR supplementation on SBP and carotid-femoral PWV (aortic stiffness) before moving towards larger-scale (phase-III) clinical trials and any recommendation of NR supplementation for improving these cardiovascular health indicators. It is also important to note that although NR is presently available as a dietary supplement under the trade name NIAGEN^®^ (ChromaDex, Inc.), the dose tested in the present study exceeds the label-recommended dose and should be considered investigational until further work can be performed to confirm the safety and efficacy of higher doses for use by the general population. Lastly, the present study assessed the influence of chronic NR supplementation on healthy middle-aged and older adults, which may have reduced the likelihood of observing greater or, in the case of several outcomes assessed, any improvements in physiological function. Thus, future investigations should include studies on groups with cardio-metabolic diseases, motor deficits, impaired NAD^+^ metabolism, and/or other disorders to determine the efficacy of NR supplementation for enhancing health status in populations with impaired baseline physiological function.

## Methods

### Ethical approval, informed consent, and study location

All procedures were approved by the University of Colorado Boulder Institutional Review Board. The nature, benefits, and risks of the study were explained to all subjects, and their written informed consent was obtained prior to participation. All measurements were performed at the University of Colorado Boulder Clinical & Translational Research Center (CTRC) and in the Integrative Physiology of Aging Laboratory. The study was registered on ClinicalTrials.gov under the identifier NCT02921659.

### Study participants

Middle-aged and older men and postmenopausal women aged 55−79 years were recruited from Boulder Colorado and surrounding communities. All subjects were free of clinical diseases, including peripheral artery disease (ankle-brachial index >0.90) and overt CVD as assessed by a graded exercise test, baseline blood panel, medical history, and physical examination by a physician. All subjects demonstrated age-related impairments in vascular endothelial function (defined as a flow-mediated dilation value <6%) and were excluded if they exhibited abnormal blood chemistries for renal or liver function (defined as 1 standard deviation outside of the normal range), had alcohol dependence, uncontrolled thyroid disease, severe obesity (body mass index >40 kg m^−2^), or were not weight stable for at least 3 months prior to enrolling in the study (defined as >2 kg change in body mass). Body mass, BMI, and waist and hip circumferences were measured by anthropometry, and total body fat percentage was measured using dual-energy x-ray absorptiometry (Lunar/Prodigy, GE). Fasting glucose and total, LDL, and HDL cholesterol levels were measured using standardized assays at the University of Colorado Boulder CTRC Core Laboratory at baseline and after each intervention phase of the study.

### Study design, randomization, and intervention

The study design consisted of a 2 × 6-week randomized, double-blind, placebo-controlled crossover clinical trial. Subjects ingested nicotinamide riboside chloride (NIAGEN^®^; 500 mg, twice per day; ChromaDex, Inc.) and placebo capsules for 6 weeks each in a randomly determined order. Subjects were randomized after providing informed consent and meeting all inclusion criteria. Randomization was performed by a member of the study team not involved in the assessment of outcomes. The study participants and members of the study team involved in the collection and analysis of outcomes were blinded to the treatment condition. Capsules were consumed with meals in the morning and evening. Subjects refrained from taking any over-the-counter medications for 48 h and prescription medications for 24 h prior to all experimental testing. All assessments were performed after a 12 h overnight fast with the exception of motor function tests, which were performed 2 h after a light meal or snack in order to ensure that subjects had enough energy to complete the testing battery. Subjects refrained from consuming alcohol or engaging in vigorous exercise for 24 h and refrained from taking study pills for at least 12 h prior to all testing sessions.

### Evaluation of safety and tolerability

Adherence to the intervention was assessed by pill count. Subjects reported to the laboratory every 2 weeks to receive a new bottle of capsules and to discuss any issues with tolerability or treatment-emergent AEs with a member of the research team who was not involved in data collection or analysis in order to ensure blinding of the investigators. Standard clinical markers of hematology, liver and kidney function and blood lipids were analyzed using standardized clinical assays at Boulder Community Hospital and any abnormal blood results were reviewed by the study physician.

### Isolation of peripheral blood mononuclear cells

PBMCs were isolated from 35 ml of whole blood collected into EDTA-coated Vacutainer™ tubes. The blood was then centrifuged at 400 × *g* for 20 min and the majority of the plasma layer (~60%) was removed to increase the efficiency of the downstream PBMC isolation. The remaining sample was slowly added to a new 50 ml conical tube containing 10 ml of Histopaque 1.077 (Greiner Bio-One) and the mononuclear cell layer was isolated by density-dependent centrifugation at 400 × *g* for 20 min, washed and then frozen in 2 ml of PBS at −80°C.

### Materials

NAAD, NAD^+^, NADP^+^, NaM, NmN, mono-, di- and triphosphate nucleotides and nucleosides were obtained from Sigma Aldrich (St. Louis, MO). Adenosine (ribose-13C5) and adenosine triphosphate (ribose-d4) were obtained from Cambridge Isotope Laboratories (Tewksbury, MA). NaM (13C6) was obtained from Cerilliant (Round Rock, TX). Nicotinamide riboside and doubly labeled nicotinamide riboside (13C1, H^2^-1) were obtained from ChromaDex Inc. (Irvine, CA). All HPLC solvents and extraction solvents were HPLC grade or better.

### Preparation of calibration standards

Individual stock standards were prepared by dissolving 10 mg ml^−^^1^ in 1:1 methanol:water and then combining to obtain a stock mixture. The NAD^+^ metabolite combined stock was prepared at 100 μg ml^−1^ of each compound and the nucleoside/nucleotide combined stock was prepared at 400 μg ml^−1^ of each compound; both were frozen at −20 °C until use. The internal standard solution was prepared at 250 μg/ml adenosine-13C5, adenosine triphosphate-d4, doubly labeled nicotinamide riboside and 2.5 μg ml^−1^ of NaM-13C6 in 1:1 methanol:water. Immediately before analysis of each sample batch, the individual calibration curve standards were prepared by combining the NAD^+^ and nucleoside/nucleotide stocks at a ratio of 1:1 and then diluting them in 1:1 methanol:water to the required concentrations. Concentrations ranged from 0.025 to 25 μg ml^−1^ for the NAD^+^ metabolites and from 0.1 to 100 μg ml^−1^ for nucleosides/nucleotides. Internal standard concentrations in all calibration levels and samples were 50 μg ml^−1^ for adenosine-13C5, adenosine triphosphate-d4 and doubly labeled nicotinamide riboside, and 0.5 μg ml^−^^1^ for NaM-13C6.

### Extraction of NAD^+^ metabolites and nucleosides/nucleotides

Frozen PBMCs (5×10E6 cells total) were thawed on ice. 500 μl of ice cold 70:30 methanol:water was added along with 20 μl of internal standard and samples vortexed for 10 s. The resulting extract was centrifuged at 8000 × *g* for 5 min at 4 °C. The resulting supernatant was transferred to a new centrifuge tube and stored on ice. To the remaining pellet, 500 μl of ice cold methanol was added and the sample vortexed for 10 s to resuspend the pellet. The sample was then centrifuged at 8000 × *g* for 5 min at 4 °C. The entire supernatant was removed and combined with the 70% methanol supernatant. The resulting pellet was reserved and frozen at −70 °C for protein concentration analysis using the Bradford assay. The combined supernatants were centrifuged at 18,000 × *g* for 15 min and the resulting supernatant was transferred to a new tube and dried in a vacuum centrifuge at 55 °C. The dried samples were reconstituted in 100 μl of 1:1 methanol:water and centrifuged at 18,000 × *g* for 10 min at 4 °C. The supernatant was then transferred to a reduced surface activity autosampler vial for analysis.

### LC-MS

HPLC separation of NAD^+^ metabolites and nucleosides/nucleotides was performed using a method described by Evans et al.^59^ with minor modifications. Separation of NAD^+^ metabolites and nucleosides/nucleotides was performed on a 1200 series HPLC from Agilent (Santa Clara, CA) using a 100 × 2 mm 5 μm Luna NH2 column from Phenomenex (Torrance, CA) operated in HILIC mode. Buffer A consisted of 100% acetonitrile and buffer B consisted of 95:5 water with 20 mm ammonium acetate adjusted to pH 9.6 with 20 mm ammonium hydroxide. Ten microliters of the extracted sample was analyzed using the following gradient at a flow rate of 0.6 ml per min: linear gradient from 5 to 100% B over 6 min, hold at 100% B from 6 to 9.5 min, then 100−5% B from 9.5 to 10.5 min, followed by re-equilibration at 5% B from 10.5 to 14 min. The column temperature was held at 15 °C for the entire gradient. Mass spectrometric analysis was performed on an Agilent 6410 triple quadrupole mass spectrometer in positive ionization mode. The drying gas was 300 °C at a flow rate of 12 ml per min. The nebulizer pressure was 30 psi. The capillary voltage was 4000 V. Data for NAD^+^ metabolites and nucleosides/nucleotides were acquired in MRM mode using experimentally optimized conditions obtained by flow injection analysis of authentic standards (Supplementary Table 5). Calibration standards were analyzed over a range of concentrations from 0.25 to 250 ng on column for the NAD^+^ metabolites and from 1 to 1000 ng on column for nucleosides/nucleotides. Calibration curves for each NAD^+^ metabolite and nucleoside/nucleotide were constructed using Agilent Masshunter Quantitative Analysis software. Results for PBMCs were quantitated using the calibration curves to obtain the on-column concentration, followed by normalization of the results using the protein concentration of the pellet reserved from the PBMC extraction.

### Assessments of cardiovascular function

Resting blood pressure was measured in the seated position after at least 10 min of quiet rest using a semi-automated blood pressure device (Dynamap™ XL, Johnson & Johnson, Arlington, TX, USA). Measurements were made multiple times from the non-dominant arm, with 2 min of quiet rest between recordings. Repeat measurements were made until three blood pressure values were obtained that were within 5 mmHg of one another. These values were then averaged to determine resting systolic and diastolic blood pressure and pulse pressure. Baseline blood pressure values were obtained using the above-described protocol on two separate testing days prior to the initiation of the first intervention arm and were averaged to determine baseline blood pressure status (i.e., normal vs. above normal) for subsequent analyses.

Aortic stiffness was measured using carotid-to-femoral PWV, the gold-standard assessment of elastic artery stiffness in humans^38^. Pressure waveforms were recorded simultaneously from the carotid and femoral arteries using applanation tonometry (Millar Inc., Houston, Texas) as previously described by our laboratory^60–62^. The transit time of the aortic pulse wave was determined by measuring the time-delay between the foot of the carotid and femoral pressure waves using LabChart analysis software. PWV was calculated by dividing the distance between the two measurement sites by the aortic transit time.

Carotid artery compliance was determined by the change in diameter of the right common carotid artery (assessed using high resolution ultrasonography, PowerVision 6000, Toshiba) relative to the change in carotid blood pressure (assessed using applanation tonometry, Millar Inc., Houston, TX) across the cardiac cycle. Carotid pressure was normalized to brachial artery pressure obtained using an automated blood pressure cuff (Dynamap™ XL, Johnson & Johnson, Arlington, TX, USA). Compliance was calculated as CC = *π* × DD^2^ × (ΔD DD^−1^)/(2 × PP), where DD is diastolic diameter, ΔD is the change in diameter and PP is the arterial pulse pressure, as has been described previously^62–65^.

Endothelium dependent dilation was measured as brachial artery flow-mediated dilation (FMD) to reactive hyperemia, using high-resolution ultrasonography (PowerVision 6000, Toshiba) as previously described^66–68^. FMD was expressed as the percentage change (%Δ) from baseline diameter.

### Assessments of metabolic function

Three-day dietary records were collected at baseline and during the last week of each intervention phase to ensure stability of caloric intake. Results were analyzed by a registered dietician using the Nutrition Data System for Research (University of Minnesota) as previously described by our laboratory^67,69^.

Resting metabolic rate was measured by indirect calorimetry (ParvoMedics TrueOne 2400) as described previously by our laboratory^70,71^. Subjects rested in a supine position for 45–60 min with a ventilated hood placed over their head to collect concentrations of expired oxygen (O_2_) and carbon dioxide (CO_2_). Metabolic rate and respiratory exchange ratio (RER) were calculated in 1-min segments and averaged from at least 30 min of steady data.

Insulin sensitivity was assessed by measuring insulin-stimulated whole-body glucose uptake using a modified frequently sampled intravenous glucose tolerance test and the Minimal Model Method of analysis as described in detail elsewhere^72^. Insulin resistance and beta cell sensitivity were assessed using the homeostasis model assessment (HOMA) method as previously described^73^.

### Assessments of exercise capacity and physical function

Cardiorespiratory fitness was determined from a graded treadmill exercise test to volitional exhaustion using a modified Balke protocol as previously described^74^. Oxygen consumption (VO_2_) and RER were measured using open-circuit spirometry with an online, computer-assisted analysis system. Heart rate and ratings of perceived exertion (RPE) were also measured throughout the test.

Walking endurance was assessed by measuring the distance covered during a 6-min walking task on a 50-foot (out-and-back) indoor course as previously described^75^.

Muscle strength and rate of torque development were quantified by measuring the peak force produced during a maximal voluntary contraction, and rate of torque development was measured using the maximal rate of developing torque during a rapid, forceful contraction of the knee flexor and extensor muscles as previously described by our laboratory^76^. Handgrip strength was measured using a standard handgrip dynamometer.

Leg fatigability was assessed using performance until failure during a single-leg heel-rise task. Subjects were asked to perform one complete plantar flexion contraction every 2 s until failure, and the test was terminated when the subject voluntarily stopped due to discomfort or inability to achieve at least 50% of maximal plantar flexion without using upper extremities for more than balance^77^. The chronic attribute of fatigue was also assessed using a Fatigue Questionnaire and Fatigue Severity Scale^78^.

Dynamic balance was assessed using a rapid step test. Maximal step length was measured in each direction (forward, backward, left, right), and targets were placed on the ground at 80% of the subject’s maximum with lines of colored tape, as described previously^79^. Performance was quantified as the time taken and number of errors committed on average during three rounds of the rapid step balance test. Each round consisted of 18 commands instructing subjects to step with a random foot to a random direction (i.e. left front). An error was defined as failure to completely step beyond the target, loss of balance, failure to return to the initial starting position, taking multiple steps to completely reach a target, or stepping with the incorrect leg or to the wrong target.

Mobility was assessed as the time to complete a 4-m walk task (performed in duplicate at the subjects' preferred walking speed) and the five-repeated sit-to-stand test (performed in triplicate), as previously described^75,78,80^. The test involves rising from a seated position in a standard-height chair five times, as quickly as possible, without using their arms for momentum or support.

Manual dexterity was assessed as the time to complete a 9-hole pegboard test as previously described^76^. Subjects collected smooth, rounded pegs from a dish and placed them into a pegboard, then returned them to the dish as quickly as possible. Two trials were completed with each hand.

### Statistical analyses

The sample size for this study was sufficient to detect at least a 50% increase in NAD^+^ concentration following NR supplementation vs. placebo (effect size = 0.7; mean of difference = 7; 1−*β* = 0.8; *α* = 0.05) as well as a clinically relevant improvement in the cardiovascular parameter with the lowest effect size (FMD; mean difference = >1%; effect size = 0.86). Estimate of effect size for NAD^+^ was determined from preliminary data of NAD^+^ metabolite concentrations in PBMCs collected from human subjects. Effect size for FMD was determined from our laboratory’s previous crossover interventions demonstrating improvements in vascular function^67^. The required sample size was determined to be 19 subjects. Assuming a 20% dropout (4 subjects) and 40% exclusion due to screen failures (consistent with other intervention studies in our laboratory using dietary supplements^81,82^), a total of 60 participants were consented for this study. Significance was set at *α* = 0.006 for all secondary outcomes to adjust for multiple testing of NR vs. placebo (paired *t-*tests) on each of the following nine pre-specified hypotheses: (1) NAD^+^ metabolites, (2) cardiovascular parameters, (3) hematology, (4) metabolic panel, (5) lipid profiles, (6) energy balance, (7) glycemic control, (8) motor function and (9) exercise performance. Because many of the measures within each hypothesis are correlated with one another (e.g., cardiovascular measures, Supplemental Table 9), each group of measures listed above was treated as one outcome when adjusting for multiple comparisons. With the exception of our primary outcome variables (NAD^+^ metabolites: NAD^+^, NAAD, NADP, NaM, NMN), in which inferences were based on an unadjusted alpha level set at 0.05, all inferences of significance are based on the Bonferroni-adjusted alpha level (*α* = 0.006).

The intent of this study was to translate promising preclinical evidence for the efficacy of chronic supplementation with NAD^+^ boosting compounds to humans. Therefore, each outcome was tested under a directional hypothesis that was determined a priori, based upon previous studies reported in the literature. Accordingly, one-tailed hypothesis tests were used to compare the proposed unidirectional effects of NR supplementation vs. placebo on these outcomes. This method has been recommended elsewhere for Phase I and II placebo-controlled clinical trials in which the goal is to gain early insight into the potential efficacy of a compound^83^.

Prior to analysis, all continuous outcome variables were assessed for normality using the Shapiro−Wilk test and by examining individual frequency histograms for each outcome. If a variable was non-normally distributed, it was log-transformed prior to analysis. If log-transformation did not normalize the data, treatment condition was analyzed using the non-parametric Wilcoxon signed rank test. For each variable, any subject with a missing value during either phase was excluded from that analysis. Based on the interpretation of the primary data, post-hoc analyses were performed to compare the change in blood pressure and aortic stiffness between subjects who exhibited normal vs. above normal baseline blood pressure using an un-paired two-tailed *t-*test. We also explored the relation between baseline NAD^+^ concentrations and the overall increase in NAD^+^ using a Pearson correlation. A formal washout period was not included in the study design; however, given the crossover design, we tested for presence of a carryover effect for each of the outcomes under study using linear regression modeled with an indicator for treatment order (no carryover effects were observed between conditions). All statistical analyses were performed using the R statistical computing platform (version 3.2.2) and GraphPad Prism 7 software.

### Data availability

The data that support the findings of this study are available from the corresponding author upon reasonable request. This trial is registered on ClinicalTrials.gov under the identifier NCT02921659.

## Electronic supplementary material

Supplementary Information
