# Supplementary material for: Chronic nicotinamide riboside supplementation is well-tolerated and elevates NAD+ in healthy middle-aged and older adults
Source: Nat Commun. 2018 Mar 29;9:1286. doi: 10.1038/s41467-018-03421-7 (PMC5876407; doi:10.1038/s41467-018-03421-7)
Supplement: Supplementary file 1 — Supplementary Information [file 41467_2018_3421_MOESM1_ESM.pdf]

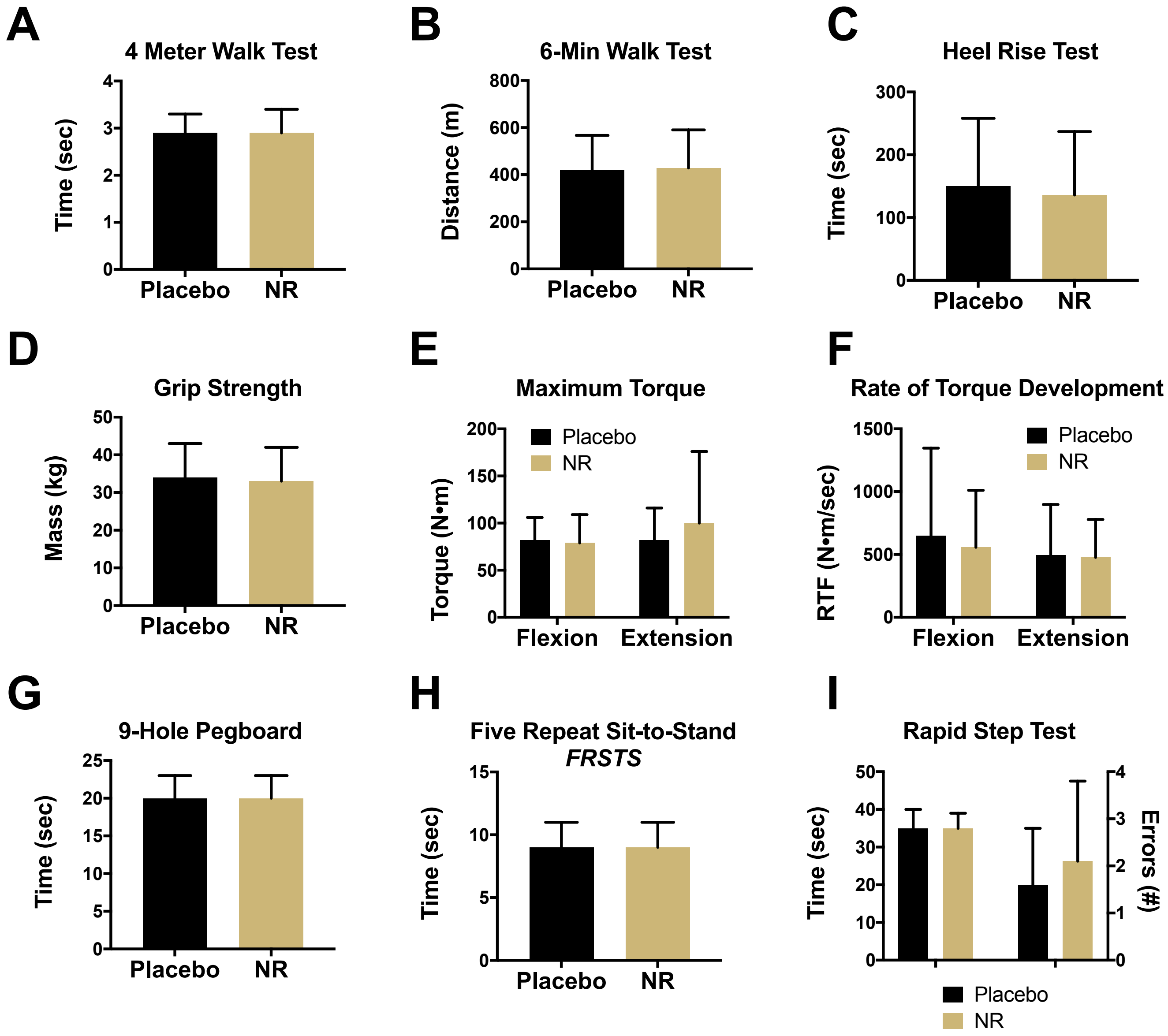

Supplementary Figure 1. Effect of 6 weeks of oral placebo vs. NR supplementation on measures of motor function. Data are mean ± SD.

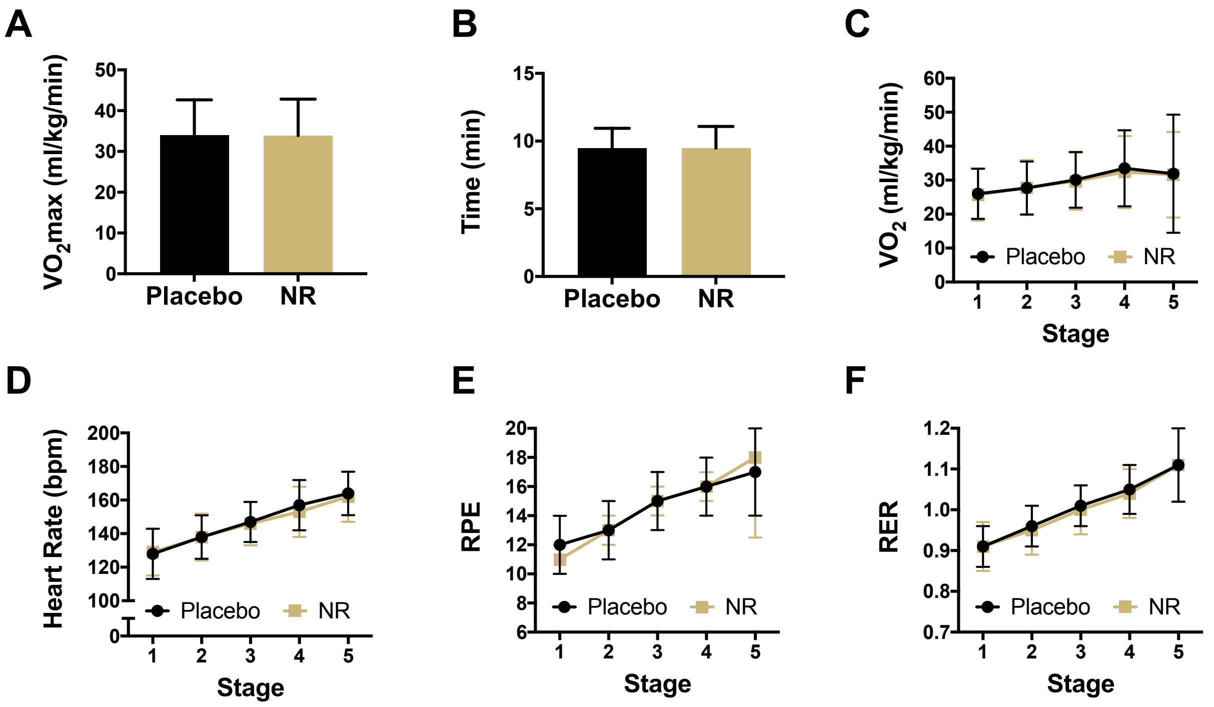

Supplementary Figure 2. Effect of 6 weeks of oral placebo vs. NR supplementation on (A) maximal oxygen uptake, (B) treadmill time to exhaustion and submaximal measures of exercise performance including (C) oxygen uptake, (D) heart rate, (E) ratings of perceived exertion and (F) respiratory exchange ratio (RER) during a graded exercise test on a treadmill. Data are mean ± SD. N = 21.

Supplementary Table 1. Complete blood count with differential collected after 6 weeks of oral placebo vs. NR supplementation with standard reference range. Data are mean ± SD. N = 21 subjects.

	**Placebo**	**NR**	**Reference Range**
HGB (g dL^{-1})	13.8 ± 1.2	13.8 ± 1.1	12.6 – 16.3 g dL^{-1}
HCT (%)	40.9 ± 3.2	40.9 ± 3.2	38.0 – 47.0 %
WBC (10^3 μL^{-1})	4.5 ± 1.2	4.3 ± 1.2	3.8 – 9.5 10^3 μL^{-1}
RBC (10^6 μL^{-1})	4.5 ± 0.4	4.4 ± 0.4	4.18 – 5.33 $10^6 \mu L^{-1}$
RDW (%)	12.9 ± 0.6	13.0 ± 0.7	11.5 – 15.2 %
MCV (fL)	92.1 ± 4.6	92.3 ± 4.1	81.5 – 99.8 fL
MCH (pg)	31.1 ± 1.4	31.2 ± 1.5	27.9 – 34.1 pg
MCHC (g dL^{-1})	33.8 ± 0.7	33.8 ± 0.9	32.4 – 36.7 g dL^{-1}
MPV (fL)	10.8 ± 1.0	10.6 ± 1.0	8.7 – 11.7 fL
Platelet count (10^3 μL^{-1})	198 ± 50	206 ± 65	150 – 400 10^3 μL^{-1}
Lymphocytes			
Relative (%)	29.5 ± 6.7	29.5 ± 7.6	15.0 – 45.0 %
Absolute (10^3 μL^{-1})	1.3 ± 0.4	1.2 ± 0.4	1.00 – 3.00 10^3 μL^{-1}
Monocytes			
Relative (%)	10.2 ± 2.1	10.6 ± 2.5	4.5 – 13.0 %
Absolute (10^3 μL^{-1})	0.5 ± 0.1	0.5 ± 0.2	0.30 – 0.80 10^3 μL^{-1}
Neutrophils			
Relative (%)	55.9 ± 6.0	55.3 ± 6.6	39.3 – 74.2 %
Absolute (10^3 μL^{-1})	2.5 ± 0.8	2.4 ± 0.8	1.70 – 6.50 10^3 μL^{-1}
Eosinophils			
Relative (%)	3.5 ± 2.4	3.1 ± 2.2	0.6 – 7.6 %
Absolute (10^3 μL^{-1})	0.2 ± 0.2	0.1 ± 0.1	0.03 – 0.40 10^3 μL^{-1}
Basophils			
Relative (%)	0.9 ± 0.3	1.2 ± 1.0	0.3 – 1.7 %
Absolute (10^3 μL^{-1})	0.04 ± 0.02	0.05 ± 0.03	0.02 – 0.10 10^3 μL^{-1}

Data are mean ± standard deviation (SD); HGB, hemoglobin; HCT, hematocrit; WBC, white blood count; RBC, red blood count; RDW, red cell distribution width; MCV, mean corpuscular volume; MCH, mean corpuscular hemoglobin; MCHC, mean corpuscular hemoglobin concentration; MPV, mean platelet volume. *$P < 0.05$ vs. placebo.

Supplementary Table 2. Comprehensive metabolic panel collected after 6 weeks of oral placebo vs. NR supplementation with standard reference range. Data are mean ± SD. N = 21 subjects.

	**Placebo**	**NR**	**Reference Range**
Total protein (g dL^{-1})	6.3 ± 0.5	6.3 ± 0.5	6.3 – 8.2 g dL^{-1}
Albumin (g dL^{-1})	3.7 ± 0.3	3.6 ± 0.3	3.5 – 5.0 g dL^{-1}
AST (SGOT) (IU L^{-1})	33 ± 13	32 ± 7	14 – 46 IU L^{-1}
Alk phosphatase (IU L^{-1})	64 ± 15	63 ± 13	38 – 126 IU L^{-1}
Total bilirubin (mg dL^{-1})	0.8 ± 0.4	0.8 ± 0.4	0.1 – 1.4 mg dL^{-1}
ALT (SGPT) (IU L^{-1})	32 ± 8	31 ± 11	9 – 52 IU L^{-1}
Creatinine (mg dL^{-1})	0.9 ± 0.2	0.9 ± 0.2	0.6 – 1.0 mg dL^{-1}
BUN (mg dL^{-1})	18 ± 5	17 ± 4	7 – 23 mg dL^{-1}
eGFR (ml/min 1.73m^{-2})	75 ± 14	74 ± 14	≥ 60 mg dL^{-1}
Calcium (mg dL^{-1})	9.2 ± 0.3	9.2 ± 0.3	8.5 – 10.4 mg dL^{-1}
Glucose (mg dL^{-1})	87 ± 7	87 ± 7	70 – 100 mg dL^{-1}
Sodium (mEq L^{-1})	139 ± 2	139 ± 2	134 – 144 mEq L^{-1}
Potassium (mEq L^{-1})	4.2 ± 0.3	4.2 ± 0.3	3.5 – 5.2 mEq L^{-1}
Chloride (mEq L^{-1})	104 ± 3	104 ± 3	97 – 110 mEq L^{-1}
CO_2 (mEq L^{-1})	26 ± 3	26 ± 3	22 – 31 mEq L^{-1}

Data are mean ± standard deviation (SD); AST, aspartate aminotransferase; SGOT, serum-oxaloacetic transaminase; alk, alkaline; ALT, alanine aminotransferase; SGPT, serum glutamic-pyruvic transaminase; BUN, blood urea nitrogen; eGFR, estimated glomerular filtration rate; CO_2, carbon dioxide.

Supplementary Table 3. Total lipid panel collected after 6 weeks of oral placebo vs. NR supplementation with standard reference range. Data are mean ± SD. N = 21 subjects.

	**Placebo**	**NR**	**Reference Range**
Total cholesterol (mg dL^{-1})	167 ± 24	171 ± 29	140 – 220 mg dL^{-1}
Triglycerides (mg dL^{-1})	90 ± 74	90 ± 62	35 – 135 mg dL^{-1}
HDL-C (mg dL^{-1})	60 ± 20	60 ± 21	40 – 85 mg dL^{-1}
LDL-C (mg dL^{-1})	90 ± 20	94 ± 25	80 – 100 mg dL^{-1}
VLDL-C (mg dL^{-1})	18 ± 15	18 ± 12	8 – 25 mg dL^{-1}
N-HDL-C (mg dL^{-1})	108 ± 27	111 ± 30	90 – 129 mg dL^{-1}

Data are mean ± standard deviation (SD); HDL-C, high-density lipoprotein cholesterol; LDL-C, low-density lipoprotein cholesterol; VLDL-C, very low-density lipoprotein cholesterol; N-HDL-C, non-HDL cholesterol.

Supplementary Table 4. Comprehensive nucleotide metabolite concentrations in peripheral blood mononuclear cells after oral placebo vs. NR supplementation normalized to total protein content. Data are mean ± SD. N=21.

Metabolite (pMol per mg protein)	Placebo	NR
NaM	277.4 ± 72.8	383.8 ± 282.7
Adenosine	10.1 ± 4.8	14.4 ± 9.7 *
Uridine	128.0 ± 60.2	278.3 ± 718.4
NR	< LOQ	< LOQ
Cytidine	2.9 ± 3.7	4.7 ± 6.6
Guanosine	1.5 ± 5.4	0.5 ± 1.5
2-3 cAMP	0.0 ± 0.0	0.0 ± 0.0
3-5 cAMP	0.0 ± 0.0	0.0 ± 0.0
cCMP	0.0 ± 0.0	1.8 ± 8.4
NAD+	10.1 ± 6.7	16.2 ± 14.1 *
NMN	0.5 ± 1.3	1.2 ± 3.3
cGMP	0.0 ± 0.0	0.0 ± 0.0
NAAD	0.2 ± 0.6	1.4 ± 2.2 *
FAD	9.9 ± 4.6	10.2 ± 5.9
CMP	48.8 ± 25.1	137.2 ± 366.0
AMP	269.6 ± 132.8	770.8 ± 1884.3
UMP	551.2 ± 243.7	1113.7 ± 2374.2
GMP	513.9 ± 195.5	1010.1 ± 2114.8
NADP	7.7 ± 4.0	8.9 ± 8.7
CDP	34.8 ± 16.0	67.7 ± 113.1
UDP	151.2 ± 79.5	270.0 ± 420.7
ADP	566.2 ± 231.6	1133.8 ± 1880.4
GDP	115.3 ± 51.0	198.4 ± 329.0
CTP	22.6 ± 12.4	41.0 ± 68.4
UTP	276.1 ± 123.5	340.3 ± 150.1
ATP	1736.3 ± 861.7	2435.7 ± 1230.3
GTP	279.5 ± 114.2	327.1 ± 119.1

* $p < 0.05$. Data are mean ± SD. N = 21.

Supplementary Table 5. Acquisition parameters used for the LC/MS/MS analysis of nucleotides, nucleosides and NAD^+ metabolites. Optimal parameters were determined by flow injection analysis of authentic standards.

Compound name	Transition	Collision Energy	Retention time	Associated internal standard
NaM (niacinamide)	123.1 -> 80.0	20	0.79	Nicotinamide 13C6
Adenosine	268.1 -> 136.1	16	2.21	Adenosine (ribose-13C5)
Uridine	245.1 -> 113.0	8	2.27	Adenosine (ribose-13C5)
Nicotinamide riboside	255.1 -> 123.1	8	2.53	Nicotinamide riboside-d2
Cytidine	244.1 -> 112.1	8	2.60	Adenosine (ribose-13C5)
Guanosine	284.1 -> 152.1	8	3.18	Adenosine (ribose-13C5)
2,3-cAMP	330.1 -> 136.1	20	3.93	ATP (ribose-d4)
3,5-cAMP	330.1 -> 136.1	24	4.09	Adenosine (ribose-13C5)
cCMP	306.1 -> 112.1	20	4.47	ATP (ribose-d4)
NAD+	664.1 -> 136.0	56	4.63	ATP (ribose-d4)
NmN	335.1 -> 123.0	12	4.83	ATP (ribose-d4)
cGMP	346.1 -> 152.1	20	5.11	ATP (ribose-d4)
NAAD	665.1 -> 136.0	56	5.24	ATP (ribose-d4)
FAD	786.2 -> 136.1	44	5.47	ATP (ribose-d4)
CMP	324.1 -> 112.1	8	5.54	Adenosine (ribose-13C5)
AMP	348.1 -> 136.1	16	5.64	Adenosine (ribose-13C5)
UMP	325.1 -> 97.0	12	5.67	Adenosine (ribose-13C5)
GMP	364.1 -> 152.1	12	6.12	Adenosine (ribose-13C5)
NADP+	744.1 -> 136.0	72	6.30	ATP (ribose-d4)
CDP	404.0 -> 112.1	16	6.52	Adenosine (ribose-13C5)
UDP	405.0 -> 97.1	16	6.73	Adenosine (ribose-13C5)
ADP	428.0 -> 136.1	28	6.73	Adenosine (ribose-13C5)
GDP	444.0 -> 152.1	20	7.20	Adenosine (ribose-13C5)
CTP	484.0 -> 112.1	20	7.36	Adenosine (ribose-13C5)
UTP	485.0 -> 97.1	24	7.55	ATP (ribose-d4)
ATP	508.0 -> 410.0	60	7.68	ATP (ribose-d4)
GTP	524.0 -> 135.0	16	8.34	ATP (ribose-d4)
Nicotinamide 13C6 (internal standard)	129.1 -> 85.1	24	0.78	-
Adenosine ribose-13C5 (internal standard)	273.1 -> 136.0	16	2.22	-
Nicotinamide riboside-d2 (internal standard)	257.1 -> 124.1	8	2.56	-
ATP ribose-d4 (internal standard)	512.0 -> 136.0	36	7.65	-

Supplementary Table 6. Median and range for systolic blood pressure (SBP), diastolic blood pressure (DBP), pulse pressure (PP), pulse wave velocity (PWV), carotid compliance (CC) and flow-mediated dilation (FMD) in all subjects at each time point (N = 24).

Outcome	Median		Range	
	**Placebo**	**NR**	**Placebo**	**NR**
SBP (mmHg)	123	115	87-157	85-143
DBP (mmHg)	71	70	55-87	51-90
PP (mmHg)	51	45	28-79	30-71
PWV (cm sec^{-1})	791	796	581-1166	536-1009
CC (mm^2 $mmHg^{-1}$ x 10^{-2})	0.074	0.081	0.029-0.272	0.021-0.202
FMD (%)	4.1	4.1	1.0-7.0	2.0-8.8

Supplementary Table 7. Effect of NR on body composition and metabolic function. Data are mean ± SD. 1 G is equivalent to the acceleration of gravity (9.8 m/s^2). Blood values missing for 3 subjects (N = 21); IVGTT performed in a subset of subjects (N=13); all other measures (N = 24).

	Placebo	NR	Sample Size (N)
Anthropometry			
Body Mass (kg)	69 ± 15	69 ± 15	24
BMI (kg m^{-2})	24 ± 5	24 ± 5	24
Total body fat (%)	28.0 ± 10.1	27.6 ± 10.0	23
Energy Intake and Expenditure			
Energy Intake (Kcal day^{-1})	1,991 ± 465	1,937 ± 486	17
Physical Activity (1x10^{-3} G day^{-1})	319 ± 125	301 ± 119	19
Resting Metabolic Rate (Kcal day^{-1})	1,417 ± 230	1,438 ± 218	22
Respiratory Exchange Ratio (RER)	0.81 ± 0.04	0.81 ± 0.04	22
Glucose/Insulin Function			
Fasting Glucose (mg dL^{-1})	93 ± 9	93 ± 9	21
Fasting Insulin (mg dL^{-1})	6.9 ± 2.4	7.0 ± 2.7	21
HOMA-IR	1.6 ± 0.6	1.6 ± 0.6	21
HOMA-β	88.3 ± 36.5	85.9 ± 34.8	21
Insulin Sensitivity (IVGTT)	4.53 ± 3.72	3.82 ± 1.88	13

Supplementary Table 8. Characteristics of subjects who dropped out of the study compared with those who completed and all subjects randomized. Data are mean ± SD.

**Subject Characteristic**	***Dropouts***	***Completers***	**All Subjects**
Sex (M/F)	*2/4*	*5/7*	**13/17**
Age (years)	*65 ± 5*	*65 ± 7*	**65 ± 7**
Mass (kg)	*73 ± 12*	*68 ± 15*	**69 ± 14**
BMI (kg/m^2)	*26 ± 3*	*24 ± 4*	**24 ± 4**
Total body fat (%)	*37 ± 9*	*28 ± 10*	**30 ± 10**
Systolic Blood Pressure (mm Hg)	*126 ± 23*	*121 ± 17*	**122 ± 18**
Diastolic Blood Pressure (mm Hg)	*74 ± 14*	*74 ± 10*	**74 ± 11**
Fasting Glucose (mg dL^{-1})	*87 ± 4*	*88 ± 8*	**88 ± 7**
Total Cholesterol (mg dL^{-1})	*225 ± 55*	*187 ± 36*	**195 ± 43**
HDL Cholesterol (mg dL^{-1})	*58 ± 17*	*69 ± 19*	**67 ± 19**
LDL Cholesterol (mg dL^{-1})	*141 ± 40*	*101 ± 30*	**109 ± 36**

Supplementary Table 9. Pair-wise correlation of primary cardiovascular outcomes (combined post-NR and post-placebo data). Data represent Pearson correlation coefficient with P-value in parentheses.

	**SBP**	**DBP**	**PP**	**PWV**	**CC**	**FMD**
**SBP**		0.74 *($1.7x10^{-9}$)*	0.87 *($6.7x10^{-16}$)*	0.54 *(0.0001)*	-0.45 *(0.003)*	0.23 *(0.137)*
**DBP**	0.74 *($1.7x10^{-9}$)*		0.32 *(0.027)*	0.41 *(0.0052)*	-0.41 *(0.007)*	0.29 *(0.067)*
**PP**	0.87 *($6.7x10^{-16}$)*	0.32 *(0.027)*		0.46 *(0.0014)*	-0.34 *(0.029)*	0.10 *(0.520)*
**PWV**	0.54 *(0.0001)*	0.41 *(0.005)*	0.46 *(0.0014)*		-0.19 *(0.252)*	0.07 *(0.640)*
**CC**	-0.45 *(0.0026)*	-0.41 *(0.007)*	-0.34 *(0.029)*	-0.19 *(0.252)*		0.02 *(0.900)*
**FMD**	0.23 *(0.137)*	0.29 *(0.067)*	0.10 *(0.520)*	0.07 *(0.640)*	0.02 *(0.900)*	
